# Laparoscopic treatment of celiac axis compression by the median arcuate ligament and endovascular repair of a pancreaticoduodenal artery aneurysm: case report

**DOI:** 10.1590/1677-5449.000118

**Published:** 2018

**Authors:** Marcio Miyamotto, Cecilia Naomi Kanegusuku, Carla Mariko Okabe, Christiano Marlo Paggi Claus, Fernanda Zandavalli Ramos, Ágata Rothert, Ana Paula Nudelmann Gubert, Ricardo César Rocha Moreira

**Affiliations:** 1 Pontifícia Universidade Católica do Paraná – PUC-PR, Hospital Universitário Cajuru – HUC, Serviço de Cirurgia Vascular e Endovascular, Curitiba, PR, Brasil.; 2 Instituto VESSEL de Aperfeiçoamento Endovascular de Curitiba, Curitiba, PR, Brasil.; 3 Hospital Nossa Senhora das Graças – HNSG, Serviço de Cirurgia Vascular e Endovascular Elias Abrão, Curitiba, PR, Brasil.; 4 Pontifícia Universidade Católica do Paraná – PUC-PR, Hospital Universitário Cajuru – HUC, Liga Acadêmica de Medicina Vascular – LAMEV, Curitiba, PR, Brasil.; 5 Hospital Nossa Senhora das Graças – HNSG, Serviço de Cirurgia Geral, Curitiba, PR, Brasil.; 6 Hospital Santa Cruz, Serviço de Cirurgia Vascular, Curitiba, PR, Brasil.

**Keywords:** median arcuate ligament syndrome, pancreaticoduodenal artery aneurysm, celiac plexus compression

## Abstract

Compression of the celiac axis by the median arcuate ligament of the diaphragm can cause nonspecific symptoms such as abdominal pain, vomiting, and weight loss. There is a known association between stenosis or occlusion of the celiac trunk and aneurysms of the pancreaticoduodenal artery. Treatment strategies for patients who have this association should be selected on a case-by-case basis. We describe the case of a patient with pancreaticoduodenal artery aneurysm associated with compression of the celiac trunk by the arcuate ligament, which were managed with endovascular and laparoscopic techniques, respectively.

## INTRODUCTION

 The median arcuate ligament is formed by fibrous bands that connect the right and left crura of the diaphragm around the aortic hiatus. The ligament may exert extrinsic compression on the celiac trunk if its location is low, or if the origin of the vessel is high. 1

 The association between stenosis or occlusions of the celiac trunk (irrespective of whether or not they are caused by extrinsic compression by the arcuate ligament) and aneurysms of the pancreaticoduodenal arcade is well defined in the literature. 2 Regardless of the association, these aneurysms account for less than 2% of all visceral aneurysms. It is estimated that 63 to 80% of patients with pancreaticoduodenal artery aneurysm have stenosis or occlusion of the celiac trunk 3 and the majority of these aneurysms (around 80%) are diagnosed after rupture. 4

 We describe the case of a patient with saccular pancreaticoduodenal artery aneurysm associated with stenosis of the celiac trunk secondary to compression by the median arcuate ligament. 

## CASE DESCRIPTION

**Figure 1:**
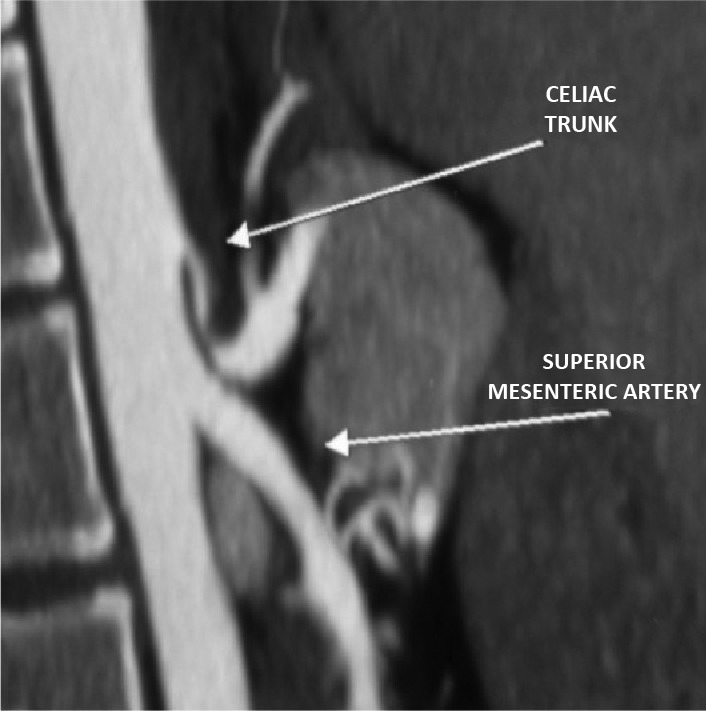
Angiotomography showing a pancreaticoduodenal artery aneurysm and compression of the origin of the celiac trunk by the arcuate ligament of the diaphragm, causing stenosis exceeding 90%.

 A 39-year-old woman with hepatitis C was being seen by the gastroenterology service to monitor a liver nodule. Abdominal ultrasonography identified a visceral artery aneurysm as an incidental finding. Angiotomography revealed that it was a saccular aneurysm of the pancreaticoduodenal artery, with a diameter of 40 mm, and showed subocclusive stenosis of the celiac trunk compatible with extrinsic compression ( Figure 1 ). 

**Figure 2:**
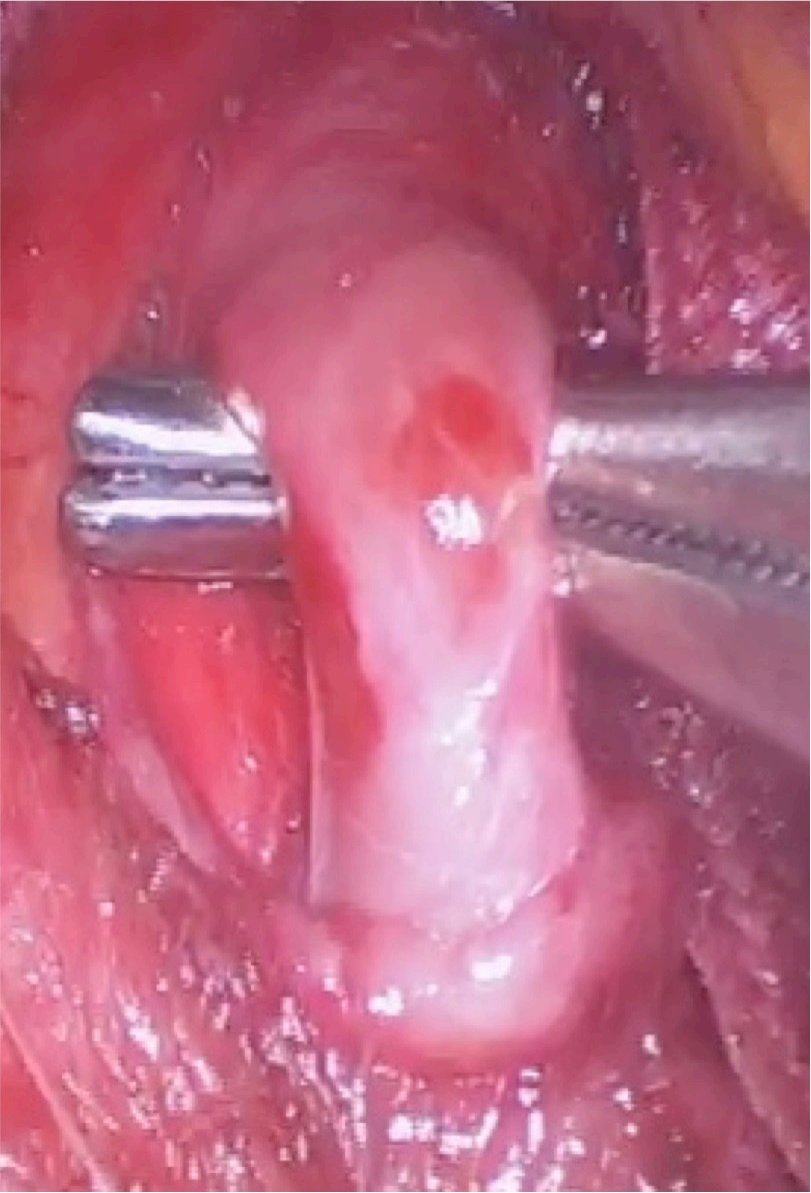
Relieving compression of the celiac trunk by sectioning the arcuate ligament via videolaparoscopy.

**Figure 3:**
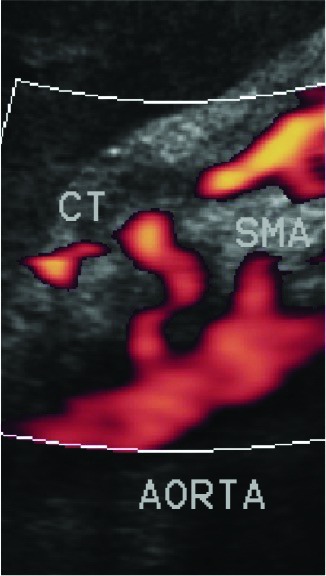
Doppler ultrasonography conducted after sectioning the arcuate ligament, showing absence of compression of the celiac trunk, leaving only residual stenosis with post-stenotic dilation. CT = celiac trunk; SMA = superior mesenteric artery.

 The patient underwent laparoscopic relief of celiac trunk compression ( Figure 2 ), thereby averting the possibility of mesenteric ischemia, as the pancreaticoduodenal artery is an important collateral route between the celiac trunk and the superior mesenteric artery and an undiscovered occlusion of this artery can cause visceral ischemia. The laparoscopic procedure was performed using a 10 mm trocar for the camera, in an umbilical position, and a further four trocars; in the right and left hypochondrium, the left flank, and a subxiphoid position. The gastrohepatic ligament, phrenoesophageal membrane, esophagus, and crura of the diaphragmatic were dissected, with inferior sectioning of the crura to enable the arcuate ligament to be viewed. Relief of celiac trunk compression was achieved by sectioning the arcuate ligament by electrocautery and the crura were drawn back together to prevent gastroesophageal reflux. Doppler ultrasonography conducted before hospital discharge showed that there was no longer compression of the celiac trunk and revealed some residual stenosis and post-stenotic dilation (the pre-stenotic celiac trunk diameter was 10 mm and at the stenosis it was 3.5 mm) ( Figure 3 ). 

**Figure 4:**
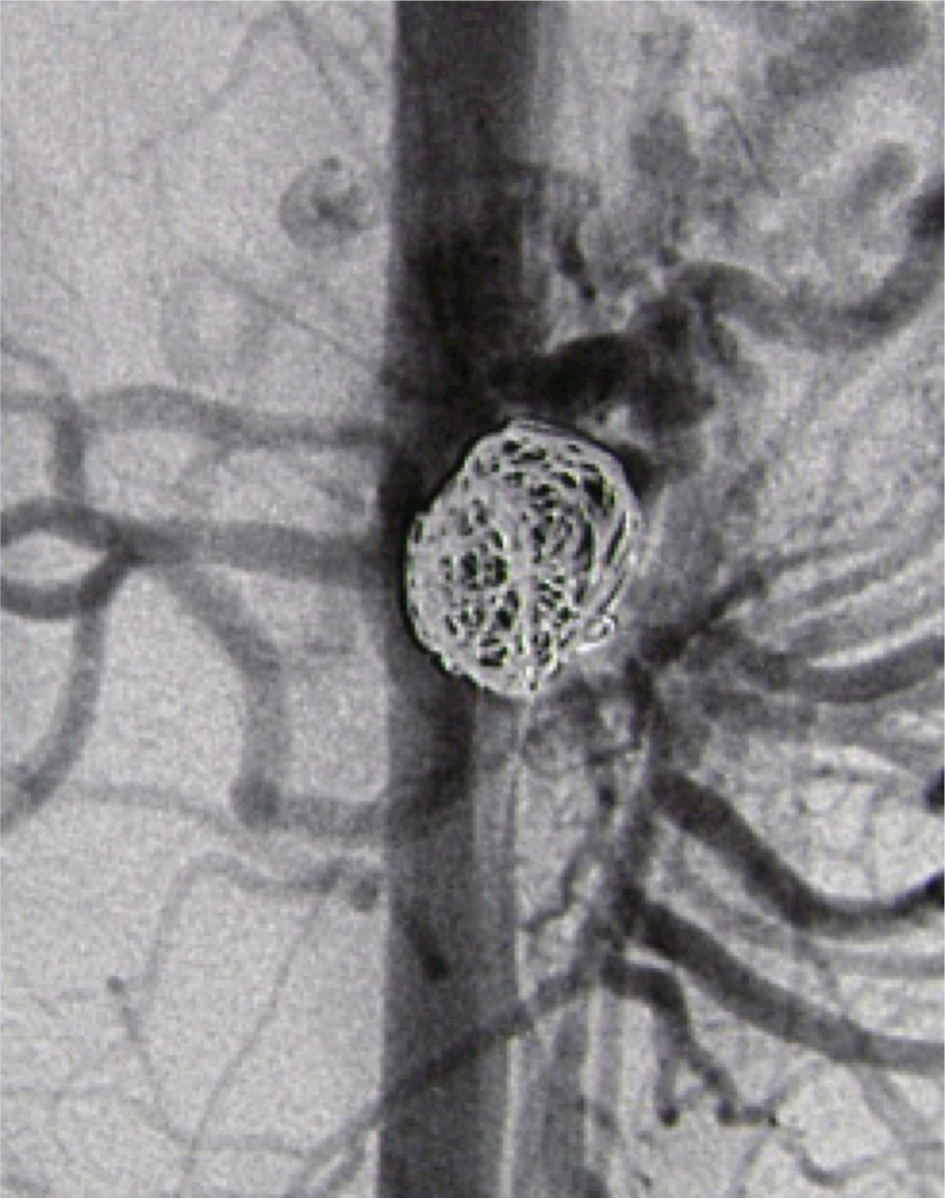
Embolization of the pancreaticoduodenal artery aneurysm with controlled release coils.

 The patient returned 2 months later for pancreaticoduodenal artery aneurysm repair, which was performed under local anesthesia and sedation, via a left brachial access with selective catheterization of the superior mesenteric artery and selective embolization of the aneurysm sac with microcoils, with no intercurrent conditions ( Figure 4 ). Four 20 mm to 25 mm x 50 cm Axium 3D microcoils and two Axium Helical microcoils 18 mm x 40 cm and 12 mm x 40 cm were used. Follow-up Doppler ultrasonography after 3 months showed thrombosis of the aneurysm and a patent pancreaticoduodenal artery, in addition to absence of extrinsic compression of the celiac trunk. 

## DISCUSSION

 Compression of the celiac trunk by the median arcuate ligament is not an uncommon situation, but arcuate ligament syndrome is a rare entity with varied and nonspecific clinical presentation, so diagnosis is by exclusion. 1 One of the first descriptions of compression of the celiac trunk by the arcuate ligament was in 1917, observed during cadaveric dissections. 1 ^,^ 5 In 1963, the syndrome was described in a patient whose symptoms were relieved after surgical section of the ligament. 6 In 1967, a series of cases of this syndrome with similar symptoms was published. 7

 Clinical presentation can include postprandial or post exercise abdominal pain, nausea, vomiting, weight loss, and epigastric bruit. 1 ^,^ 5 ^,^ 8 Occurrence of symptoms may be caused by restricted blood flow in situations of greater demand and by concomitant compression of the fibers of the periaortic celiac plexus. 1 ^,^ 8 Differential diagnosis should be conducted to rule out gastrointestinal diseases such as peptic ulcer, cholecystitis, pancreatitis, and chronic mesenteric ischemia. 

 Celiac trunk compression can be diagnosed by Doppler ultrasonography, which shows compression of the vessel and reverse flow in the hepatic artery, suggesting proximal stenosis or occlusion. An elevated systolic peak velocity in the celiac trunk only during expiration is indicative of dynamic compression. 1 ^,^ 5 ^,^ 8

 Angiography is the gold standard for diagnosis and the classic findings are an asymmetrical focal narrowing of the celiac trunk, more pronounced during expiration, with or without post-stenotic dilation. 1 ^,^ 5 Even though angiotomography is not a dynamic examination, it offers the possibility of assessing adjacent, non-vascular structures. 1

 The association between compression of the celiac trunk by the arcuate ligament (or stenosis or occlusion of any other etiology) and aneurysms of the pancreaticoduodenal artery was first described in the 1970s. 9 ^,^ 10 Pathophysiology is related to the increased blood flow through the pancreaticoduodenal arteries, 9 at the stenosis or occlusion of the celiac trunk, since flow through the territory of the superior mesenteric artery is diverted through collaterals to those with reduced flow. 1 ^,^ 8

 These aneurysms may be asymptomatic or may manifest symptoms related to extrinsic compression of the gastrointestinal or biliary tracts. 9 Intestinal bleeding can occur if the aneurysm ruptures into the duodenum and/or pancreatic ducts. 2 ^,^ 11 Diagnosis can be made by angiotomography. 6

 The risk of rupture does not appear to be related to size with these types of aneurysm. 2 The rupture-related mortality rate is high and can range from 50 to 90%. 2 ^,^ 12 Considering these two facts, there is no doubt of the need for treatment in the case described, despite the lack of a consensus on the minimum size at which treatment is indicated. 

 There is also no consensus on the need for treatment of celiac trunk compression in asymptomatic patients with pancreaticoduodenal artery aneurysms. 5 However, it seems logical that it would be necessary to relieve the compression of the celiac trunk before attempting to treat the aneurysm, in case embolization of the aneurysm is planned, in order to avoid the possibility of ischemia and recurrence of the aneurysm because the flow remains elevated. 13 However, there are no reports of recurrence of a pancreaticoduodenal artery aneurysm after embolization, even in the absence of prior treatment of the celiac trunk. 2

 Traditionally, treatment for arcuate ligament syndrome, via a midline surgical access or laparoscopy, consists of sectioning the ligament to relieve compression of the celiac trunk and eliminate irritation caused by compression of nerve fibers. 8 More recently, there has been a trend to use endovascular and laparoscopic techniques. 1

 The open procedure for treatment of this syndrome is well-documented, 14 and the patients that most benefit from the treatment are those with postprandial pain, age between 40 and 60 years, and with significant weight loss. 14 A group of 18 patients with arcuate ligament syndrome underwent open surgical treatment to section the ligament and resection of the adjacent periaortic tissues. After three and a half years of follow-up, 73.3% of the patients were asymptomatic. 5

 Treatment of arcuate ligament syndrome with videolaparoscopy was documented in 16 patients. Just two of these patients did not exhibit relief from symptoms during the postoperative period (improvement in 87.5%), because of fixed stenosis of the celiac trunk, which was managed with balloon angioplasty and stent placement. Even so, in one of these cases an aortoceliac bypass was necessary. 8

 The difficulty in treating arcuate ligament syndrome resides in the patients with nonspecific gastrointestinal symptoms. The difficulty in establishing a causal link between the anatomic condition and the presence of symptoms can result in a low level of treatment effectiveness. 

 Recent publications have demonstrated improvement in videolaparoscopic techniques, such as introduction of an ultrasonography probe, as a means of documenting the increased blood flow after the ligament is resected, and use of robots. 15 ^,^ 16 It has been demonstrated that treatment of the vascular injury in isolation does not produce good long-term results, and it is necessary to lyse the ligament fibers. 17

 As for the pancreaticoduodenal artery aneurysms, endovascular treatment tends to be indicated when the diameter exceeds two centimeters, there is rapid growth and symptoms. Other factors that should be considered are a saccular shape and location in collateralization arteries. Aneurysms that are morphologically favorable for endovascular techniques are those with narrow necks, adequate collateral flow and non-terminal vessels. Endovascular management is the preferred option for pancreaticoduodenal aneurysms. 11 There are still indications for open surgical treatment of visceral aneurysms, but the endovascular approach offers several advantages, such as being less invasive, having fewer serious complications, and enabling selective embolization. 

 Embolization can be achieved with a variety of different materials, although microcoils are the most widely used. 2 There are certain limitations related to the technique when using covered stents to exclude aneurysms, such as to the release system and difficulty of fitting in more tortuous arteries, and to the risk of intra-stent thrombosis. Use of covered stents is more appropriate in arteries with diameters exceeding six millimeters and to prevent migration of microcoils in saccular aneurysms with wide necks. 2

 It is also important to point out that endovascular treatment can be used with patients who have a ruptured aneurysm. 13 Open surgical treatment is subject to technical difficulties primarily related to access to the pancreaticoduodenal arcade and to bleeding control. These difficulties have stimulated development of endovascular techniques. Notwithstanding, there are reports of successful open treatment. 18

## CONCLUSIONS

 When a patient has both compression of the celiac trunk by the arcuate ligament and a pancreaticoduodenal artery aneurysm, the treatment of both conditions is necessary. However, it is clear that less invasive treatments such as videolaparoscopy and endovascular techniques offer advantages over open surgery, considering the morbidity and mortality related to the procedure. 
