# Supplementary material for: Laparoscopic treatment of celiac axis compression by the median arcuate ligament and endovascular repair of a pancreaticoduodenal artery aneurysm: case report
Source: J Vasc Bras. 2018 Jul-Sep;17(3):252–6. doi: 10.1590/1677-5449.000118 (PMC6326135; doi:10.1590/1677-5449.000118)
Supplement: Translation

# Translation: Tratamento laparoscópico da compressão do tronco celíaco pelo ligamento arqueado do diafragma associado a correção endovascular do aneurisma de artéria pancreatoduodenal: relato de caso

**Authors:** Marcio Miyamotto, Cecilia Naomi Kanegusuku, Carla Mariko Okabe, Christiano Marlo Paggi Claus, Fernanda Zandavalli Ramos, Ágata Rothert, Ana Paula Nudelmann Gubert, Ricardo César Rocha Moreira

**Affiliations:** 1 Pontifícia Universidade Católica do Paraná – PUC-PR, Hospital Universitário Cajuru – HUC, Serviço de Cirurgia Vascular e Endovascular, Curitiba, PR, Brasil.; 2 Instituto VESSEL de Aperfeiçoamento Endovascular de Curitiba, Curitiba, PR, Brasil.; 3 Hospital Nossa Senhora das Graças – HNSG, Serviço de Cirurgia Vascular e Endovascular Elias Abrão, Curitiba, PR, Brasil.; 4 Pontifícia Universidade Católica do Paraná – PUC-PR, Hospital Universitário Cajuru – HUC, Liga Acadêmica de Medicina Vascular – LAMEV, Curitiba, PR, Brasil.; 5 Hospital Nossa Senhora das Graças – HNSG, Serviço de Cirurgia Geral, Curitiba, PR, Brasil.; 6 Hospital Santa Cruz, Serviço de Cirurgia Vascular, Curitiba, PR, Brasil.

**Keywords:** síndrome do ligamento arqueado mediano, aneurisma de artéria pancreatoduodenal, compressão do plexo celíaco

## Abstract

A compressão do tronco celíaco pelo ligamento arqueado mediano do diafragma pode causar sintomas inespecíficos como dor abdominal, vômitos e emagrecimento. Existe uma associação comprovada entre estenoses ou oclusões do tronco celíaco e aneurismas da artéria pancreatoduodenal. Nas situações em que essa associação ocorre, a estratégia de tratamento deve ser individualizada. Relatamos o caso de uma paciente com aneurisma de artéria pancreatoduodenal associado à compressão do tronco celíaco pelo ligamento arqueado, manejados, respectivamente, por técnicas endovasculares e laparoscópicas.

## INTRODUÇÃO

 O ligamento arqueado mediano é formado por bandas fibrosas de conexão entre os pilares direito e esquerdo do diafragma ao redor do hiato aórtico. Esse ligamento pode gerar compressão extrínseca do tronco celíaco por sua implantação mais inferior ou pela origem mais alta desse vaso 1 . 

 A associação entre estenoses ou oclusões do tronco celíaco, causadas ou não pela compressão do ligamento arqueado, e aneurismas da arcada pancreatoduodenal é bem descrita na literatura 2 . Independentemente da associação, esses aneurismas representam menos de 2% de todos os aneurismas viscerais. Estima-se que 63 a 80% dos pacientes com aneurisma de artéria pancreatoduodenal têm estenose ou oclusão de tronco celíaco 3 , sendo que a maioria dos aneurismas (cerca de 80%) são diagnosticados após a ruptura 4 . 

 Relatamos o caso de paciente com aneurisma sacular de artéria pancreatoduodenal associado a estenose do tronco celíaco por compressão pelo ligamento arqueado mediano. 

## DESCRIÇÃO DO CASO

 Paciente do sexo feminino, 39 anos, portadora de hepatite C, estava em acompanhamento com a gastroenterologia devido a nódulo hepático. A ultrassonografia abdominal apresentou como achado incidental um aneurisma de artéria visceral. A angiotomografia evidenciou aneurisma sacular com diâmetro de 40 mm na artéria pancreatoduodenal e estenose suboclusiva de tronco celíaco compatível com compressão extrínseca ( Figura 1 ). 

**Figura 1:** Angiotomografia evidenciando a presença de aneurisma de artéria pancreatoduodenal e compressão da origem do tronco celíaco pelo ligamento arqueado do diafragma, gerando estenose acima de 90%.

 A paciente foi submetida previamente a liberação laparoscópica da compressão do tronco celíaco ( Figura 2 ), prevenindo assim uma eventual isquemia mesentérica, já que a artéria pancreatoduodenal é uma importante via colateral entre o tronco celíaco e a artéria mesentérica superior, e a oclusão inadvertida dessa artéria poderia ocasionar um quadro de isquemia visceral. O procedimento laparoscópico foi realizado através de um trocarte de 10 mm para câmera em posição umbilical e outros quatro trocartes no hipocôndrio direito e esquerdo, no flanco esquerdo e em posição subxifoide. Foram dissecados o ligamento gastro-hepático, a membrana frenoesofágica, o esôfago e os pilares diafragmáticos, com secção inferior desses últimos para visibilização do ligamento arqueado. A descompressão do tronco celíaco foi realizada pela secção do ligamento arqueado com eletrocautério e os pilares diafragmáticos foram reaproximados para prevenir o refluxo gastroesofágico. O eco-Doppler realizado antes da alta hospitalar demonstrou ausência de compressão do tronco celíaco, restando uma estenose residual com dilatação pós-estenótica (o tronco celíaco pré-estenose tem 10 mm e, na estenose, passa a ter 3,5 mm) ( Figura 3 ). 

**Figura 2:** Liberação da compressão do tronco celíaco através da secção do ligamento arqueado por videolaparoscopia.

**Figura 3:** Eco-Doppler realizado após a secção do ligamento arqueado mostrando a ausência de compressão do tronco celíaco, restando apenas uma estenose residual com dilatação pós-estenótica. TC = tronco celíaco; MS = mesentérica superior.

 A paciente retornou em dois meses para correção de aneurisma de artéria pancreatoduodenal, realizada sob anestesia local e sedação, através de acesso braquial esquerdo com cateterização seletiva da artéria mesentérica superior e embolização seletiva do saco aneurismático com micromolas, sem intercorrências ( Figura 4 ). Foram utilizadas quatro micromolas Axium 3D de 20 a 25 mm x 50 cm e duas Axium Helical de 18 mm x 40 cm e 12 mm x 40 cm. O seguimento com eco-Doppler em três meses evidenciou trombose do aneurisma com perviedade da artéria pancreatoduodenal, além de ausência de compressão extrínseca do tronco celíaco. 

**Figura 4:** Embolização do aneurisma de artéria pancreatoduodenal com molas de liberação controlada.

## DISCUSSÃO

 A compressão do tronco celíaco pelo ligamento arqueado mediano não é uma situação incomum, mas a síndrome do ligamento arqueado é uma entidade rara e tem uma apresentação clínica variada e inespecífica, sendo um diagnóstico de exclusão 1 . Uma das primeiras descrições da compressão do tronco celíaco pelo ligamento arqueado foi em 1917 através de dissecções cadavéricas 1 ^,^ 5 . Em 1963, a síndrome foi descrita em um paciente que apresentou alívio dos sintomas após a secção cirúrgica do ligamento 6 . Em 1967, foi publicada uma série de casos dessa síndrome com sintomas similares 7 . 

 A apresentação clínica pode incluir dor abdominal pós-prandial ou após exercícios, náusea, vômito, perda de peso e sopro epigástrico 1 ^,^ 5 ^,^ 8 . A ocorrência dos sintomas pode ser explicada pela diminuição de fluxo em situações de maior demanda e pela compressão concomitante das fibras do plexo celíaco periaórtico 1 ^,^ 8 . O diagnóstico diferencial deve ser realizado com doenças gastrointestinais como úlcera péptica, colecistite, pancreatite e isquemia mesentérica crônica. 

 O diagnóstico da compressão do tronco celíaco pode ser obtido por eco-Doppler evidenciando a compressão do vaso, além do fluxo reverso na artéria hepática, sugerindo estenose ou oclusão proximal. O achado da elevação do pico de velocidade sistólica no tronco celíaco somente durante a expiração é indicativo de compressão dinâmica 1 ^,^ 5 ^,^ 8 . 

 A angiografia é o exame diagnóstico de excelência, e os achados clássicos são o estreitamento focal assimétrico no tronco celíaco, mais relevante à expiração, associado ou não a dilatação pós-estenótica 1 ^,^ 5 . A angiotomografia, mesmo sendo um exame não dinâmico, permite avaliar estruturas não vasculares adjacentes 1 . 

 A associação entre a compressão do tronco celíaco pelo ligamento arqueado, ou qualquer estenose ou oclusão de outra etiologia, e aneurismas da artéria pancreatoduodenal foi descrita inicialmente na década de 1970 9 ^,^ 10 . A fisiopatologia está relacionada ao aumento de fluxo sanguíneo nas artérias pancreatoduodenais 9 , na estenose ou oclusão do tronco celíaco, já que o fluxo do território da artéria mesentérica superior é desviado por colaterais para suprir aquele com baixo fluxo 1 ^,^ 8 . 

 Esses aneurismas podem ser assintomáticos ou apresentar sintomas relacionados à compressão extrínseca do trato gastrointestinal ou biliar 9 . Sangramento intestinal pode ocorrer devido à ruptura do aneurisma no duodeno e/ou nos ductos pancreáticos 2 ^,^ 11 . O diagnóstico pode ser realizado por angiotomografia 6 . 

 O risco de ruptura desses tipos de aneurisma parece não estar relacionado ao seu tamanho 2 . A taxa de mortalidade associada à ruptura é alta e pode variar de 50 a 90% 2 ^,^ 12 . Considerando essas duas informações, embora não haja consenso sobre o tamanho mínimo para indicação de tratamento, no caso apresentado não há dúvidas sobre a necessidade da abordagem. 

 Não existe consenso sobre a necessidade do tratamento da compressão do tronco celíaco nos pacientes assintomáticos com aneurisma de artéria pancreatoduodenal 5 . Entretanto, parece lógico que a descompressão prévia do tronco celíaco seja necessária para permitir o tratamento do aneurisma, caso a embolização do aneurisma esteja prevista, para evitar um possível quadro isquêmico e a recorrência do aneurisma pela manutenção do hiperfluxo 13 . Entretanto, não há relatos de recorrência do aneurisma de artéria pancreatoduodenal após a embolização, mesmo sem o tratamento prévio do tronco celíaco 2 . 

 Tradicionalmente, o tratamento da síndrome do ligamento arqueado, por acesso cirúrgico mediano ou laparoscopia, consiste na secção do ligamento para descomprimir o tronco celíaco e, secundariamente, eliminar a irritação causada por compressão das fibras nervosas 8 . Recentemente, há a tendência da abordagem endovascular e laparoscópica 1 . 

 O procedimento aberto para correção da síndrome supracitada é bem documentado 14 , e os pacientes que mais se beneficiam do tratamento são aqueles com dor pós-prandial, idade entre 40 e 60 anos e perda de peso significativa 14 . Um grupo de 18 pacientes com síndrome do ligamento arqueado foi submetido ao tratamento cirúrgico aberto por secção do ligamento e ressecção dos tecidos adjacentes periaórticos. Após três anos e meio de seguimento, 73,3% dos pacientes apresentavam-se assintomáticos 5 . 

 O tratamento da síndrome do ligamento arqueado foi documentado em 16 pacientes por via videolaparoscópica. Apenas dois pacientes não apresentaram alívio dos sintomas no pós-operatório (melhora de 87,5%) devido à presença de estenose fixa no tronco celíaco, manejada com angioplastia por balão e implante de stent. Mesmo assim, em um desses casos, houve necessidade de confecção de ponte aorta-tronco celíaco 8 . 

 A grande dificuldade no tratamento da síndrome do ligamento arqueado reside no grupo de pacientes com sintomas gastrointestinais inespecíficos. A dificuldade no estabelecimento de um nexo causal razoável entre a condição anatômica e a presença dos sintomas pode resultar em um baixo índice de efetividade do tratamento. 

 Publicações recentes têm demonstrado aprimoramento na técnica videolaparoscópica, como a introdução de probe de ultrassonografia, como forma de documentar o incremento de fluxo sanguíneo após a liberação do ligamento, e a utilização da robótica 15 ^,^ 16 . Já foi demonstrado que o tratamento isolado da lesão vascular não produz bons resultados em longo prazo, sendo necessária a lise das fibras ligamentares 17 . 

 Com relação ao aneurisma de artéria pancreatoduodenal, o tratamento endovascular costuma ser indicado quando o diâmetro é maior que dois centímetros, com crescimento rápido ou sintomático. Outros fatores que devem ser considerados são o formato sacular e a localização em artérias de colateralização. Os aneurismas que são morfologicamente favoráveis à técnica endovascular são os de colo estreito, fluxo colateral adequado e vasos não terminais. O manejo endovascular é o preferencial em aneurismas pancreatoduodenais 11 . O tratamento cirúrgico aberto dos aneurismas viscerais ainda tem indicações, mas a abordagem endovascular tem diversas vantagens, como ser menos invasiva, ter complicações menos graves e permitir embolização seletiva. 

 A embolização pode ser realizada com diversos materiais, sendo a embolização com micromolas a mais utilizada 2 . O uso de stents recobertos para a exclusão dos aneurismas apresenta algumas dificuldades relacionadas à técnica, como limitações no sistema de liberação, dificuldade de acomodação em artérias mais tortuosas e risco de trombose intra-stent. A utilização de stents recobertos é mais indicada em artérias de diâmetro maior que seis milímetros e como prevenção da migração das micromolas em aneurismas saculares de colo largo 2 . 

 É importante ressaltar que o tratamento endovascular é factível em pacientes na vigência de ruptura do aneurisma 13 . O tratamento cirúrgico aberto apresenta dificuldades técnicas relacionadas principalmente ao acesso à arcada pancreatoduodenal e ao controle do sangramento. Tais dificuldades estimularam o desenvolvimento da técnica endovascular. Ainda assim, há relatos bem sucedidos da abordagem aberta 18 . 

## CONCLUSÃO

 Quando existe associação entre a compressão do tronco celíaco pelo ligamento arqueado e o aneurisma de artéria pancreatoduodenal, há necessidade do tratamento de ambas as condições. Entretanto, torna-se claro que os tratamentos menos invasivos como a videolaparoscopia e a técnica endovascular apresentam vantagens sobre a cirurgia aberta, considerando a morbimortalidade relacionada ao procedimento.
